# Bis(*N*,*N*′-dimethyl­ethylenediammonium) tris­(oxalato-κ^2^ *O* ^1^,*O* ^2^)cobaltate(II) dihydrate: an ion-pair complex

**DOI:** 10.1107/S1600536811031357

**Published:** 2011-08-27

**Authors:** Papa Aly Gaye, Adama Sy, Ibrahima Elhadj Thiam, Mohamed Gaye, Pascal Retailleau

**Affiliations:** a Département de Chimie, Faculté des Sciences et Techniques, Université Cheikh Anta Diop, Dakar, Senegal; b ICSN-CNRS, Laboratoire de Cristallochimie, 1 Avenue la Terasse, 91198 Gift sur Yvette, France

## Abstract

The Co^II^ ion in the title complex, (C_4_H_14_N_2_)_2_[Co(C_2_O_4_)_3_]·2H_2_O, is coordinated by three oxalate ions, resulting in a distorted octa­hedral geometry. Two uncoordinated water mol­ecules are present in asymmetric unit. Inter­molecular N—H⋯O and O—H⋯O hydrogen bonds between the different entities stabilize the crystal structure.

## Related literature

For related structures: see Diallo *et al.* (2008 ▶); Gaye *et al.* (2011 ▶); Hao *et al.* (2010 ▶); Kelly *et al.* (2005 ▶); Zhang *et al.*, (2009 ▶). 
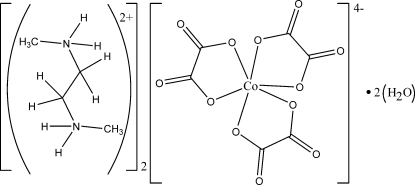


## Experimental

### 

#### Crystal data

(C_4_H_14_N_2_)_2_[Co(C_2_O_4_)_3_]·2H_2_O
*M* *_r_* = 539.37
Monoclinic, 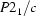

*a* = 12.625 (3) Å
*b* = 13.411 (4) Å
*c* = 16.996 (2) Å
β = 125.91 (2)°
*V* = 2330.7 (11) Å^3^
*Z* = 4
Mo *K*α radiation
μ = 0.81 mm^−1^
*T* = 293 K
0.50 × 0.48 × 0.26 mm

#### Data collection

Nonius KappaCCD diffractometer
Absorption correction: multi-scan (*SCALEPACK*; Otwinowski & Minor, 1997 ▶) *T*_min_ = 0.720, *T*_max_ = 0.800
9121 measured reflections
5342 independent reflections
4178 reflections with *I* > 2σ(*I*)
*R*_int_ = 0.018

#### Refinement

*R*[*F* ^2^ > 2σ(*F* ^2^)] = 0.047
*wR*(*F* ^2^) = 0.133
*S* = 1.05
5333 reflections
302 parameters
H-atom parameters constrained
Δρ_max_ = 1.24 e Å^−3^
Δρ_min_ = −0.48 e Å^−3^

### 

Data collection: *DENZO* (Otwinowski & Minor, 1997 ▶) and *COLLECT* (Nonius, 1999 ▶); cell refinement: *DENZO* and *COLLECT*; data reduction: *SCALEPACK* (Otwinowski & Minor, 1997 ▶); program(s) used to solve structure: *SHELXS97* (Sheldrick, 2008 ▶); program(s) used to refine structure: *SHELXL97* (Sheldrick, 2008 ▶) and *CRYSTALBUILDER* (Welter, 2006 ▶); molecular graphics: *PLATON* (Spek, 2009 ▶); software used to prepare material for publication: *SHELXL97*.

## Supplementary Material

Crystal structure: contains datablock(s) I, global. DOI: 10.1107/S1600536811031357/bt5577sup1.cif

Structure factors: contains datablock(s) I. DOI: 10.1107/S1600536811031357/bt5577Isup2.hkl

Additional supplementary materials:  crystallographic information; 3D view; checkCIF report

## Figures and Tables

**Table 1:** Hydrogen-bond geometry (Å, °)

*D*—H⋯*A*	*D*—H	H⋯*A*	*D*⋯*A*	*D*—H⋯*A*
N9—H9*N*1⋯O8	0.90	1.86	2.668 (3)	148
O14—H14*O*⋯O7	0.96	1.94	2.876 (4)	163
N7—H7*NB*⋯O1	0.90	1.90	2.769 (3)	161
O13—H13*W*⋯O10	0.96	1.91	2.755 (3)	146
N10—H10*M*⋯O11^i^	0.90	1.94	2.814 (3)	165
N10—H10*N*⋯O9^ii^	0.90	1.83	2.721 (3)	173
N9—H9*N*2⋯O13^iii^	0.90	1.80	2.679 (3)	163
N8—H8*NA*⋯O6^iv^	0.90	1.94	2.829 (3)	170
N8—H8*NB*⋯O7^v^	0.90	2.07	2.916 (3)	156
N8—H8*NB*⋯O8^v^	0.90	2.25	2.801 (3)	119
N7—H7*NA*⋯O12^vi^	0.90	1.97	2.751 (3)	144
O13—H13*O*⋯O12^vii^	0.95	1.75	2.698 (3)	174

Symmetry codes: (i) 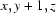; (ii) 
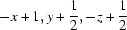
; (iii) 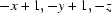; (iv) 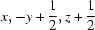; (v) 
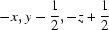
; (vi) 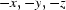; (vii) 
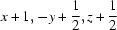
.

**Fig. 1.**
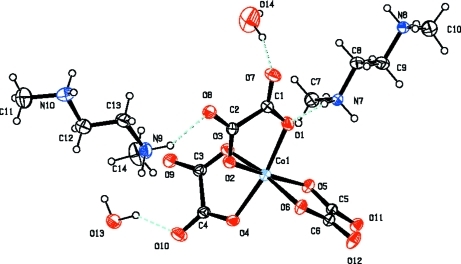
An ORTEP view of the title compound, showing the atom-numbering scheme. Displacement ellipsoids are plotted at the 50% probability level.

## supplementary crystallographic information

### Comment

The title salt, (C_4_H_14_N_2_)_2_[Co(C_2_O_4_)_3_](H_2_ O)_2_, was obtained as an unexpected product by reaction of the employed ligand (C_6_H_10_N_2_O_2_)_n_, in a methanolic medium. The hydrolytically unstable cyclic ligand apparently is oxidatively hydrolyzed in the presence of metal ions, leading to the oxalate dianion (Diallo *et al.*, 2008; Kelly *et al.*, 2005). This species, which is generated *in situ*, acts with cobat(II) ions resulting in the formation of the title compound. A similar reaction was found elsewhere (Zhang *et al.*, 2009). Recently, we published the structure of an organic-inorganic hybrid salt involving the dimethylethylenediammonium cation, [C_4_H_14_N_2_]^2+^ and complex anion, [Cu(C_2_O_4_)_2_]^2-^ (Gaye *et al.*, 2011). A homologous salt, with [Co(C_2_O_4_)_3_]^4-^ as the anionic moiety is reported here. The Fig. 1 shows the dimethylethylenediammonium cation, [C_4_H_24_N_2_]^2+^, and the complex anion, [Co(C_2_O_4_)_3_]^4-^. The asymmetric unit contains two organic cations, one anion and two water molecules. The geometrical parameters of the [C_4_H_24_N_2_]^+^, cation are similar to those found in salt with the [Cu(C_2_O_4_)_2_]^2-^ cationic complex (Gaye *et al.*, 2011). The Co^II^ ion of the complex anion adopts a distorted octahedral coordination involving four equatorial O atoms (O1, O2, O4, O5) two quasi axial O atoms (O3, O6) of oxalate ligands (Fig. 1). The equatorial Co–O distances are 2.1009 (17) Å (Co–O1), 2.0751 (18) Å (Co–O2), 2.0693 (17) (13) Å (Co–O4) and 2.0659 (17) Å (Co–O5) respectively, and are significantly longer than the axial Co–O distance of 2.1163 (19) Å (Co–O3), 2.1070 (17) Å (Co–O6). The bond distances in the complex anion are comparable with those reported for the [Co^III^(Hbiim)_3_]_2_[Co^II^_3_(ox)_3_].4H_2_O compound (Hao *et al.*, 2010), where Hbiim is 2,2'-biimidazole and ox is oxalate. Two lattice water molecules are also present in the asymmetric unit. In the crystal structure, intramolecular N–H···O_ox_ hydrogen bonds connect the ionic entities. O_water_–H···O_ox_ hydrogen bonds are also observed (Table 2).

### Experimental

In a 50 ml round bottom flask introduce Dimethyl oxalate (2.36 g, 0.020 mol) dissolved in ethanol (10 ml). *N*,*N*'-dimethyl-1,2-diaminoethane (1.77 g, 0.020 mol) in ethanol (10 ml), was added to yield immediately a quantitative precipitate. The white precipitate formed, was separated by filtration, washed with methanol and ether and dried under vacuum (Yield 3.32 g, 58.5%); m.p.=240 °C. ^1^H NMR in CDCl_3_, δ (p.p.m.): 3.1, s, 12H, –CH3; 3.5, s, 8H, –CH_2_. ^13^C NMR in CDCl_3_, δ (p.p.m.): 34.86, N—CH_3_, 46.12, N—CH_2_, 157.56, C=O. IR (cm^-1^) 1598 (C=O), 1284 (C—N). Anal. Calc. for C_12_H_20_N_4_O_4_ (%): C, 50.62; H, 7.11; N, 19.68. Found: C, 50.60; H, 7.09; N, 19.71. Mass spectrum (m/*z*) 284, 162, 134, 106, 78. Into a methanolic solution (5 ml) of cobalt chloride hexahydratee (0.2974 g, 1.25 mmol) was added a methanolic solution (10 ml) of the ligand prepared above (0.3554 g, 1.25 mmol). The resulting mixture is heated at 60°C for thirty minutes. The pink solution was filtered and then allowed to evaporate slowly in an open atmosphere. After two days, pink crystals suitable for X-ray analysis were obtained. The crystals were separated, washed with cold methanol and dried (yield: 83%); Anal. Calc. for C_14_H_32_CoN_4_O_14_(%): C, 31.18; H, 5.98; N, 10.39. Found: C, 31.16; H, 6.01; N, 10.37. Selected IR data (cm^-1^, KBr pellet): 3335, 1635, 1601, 1580, 1193, 765.

### Refinement

Nine reflections affected by the backstop or clearly outlier data were omitted from the refinement. All H atoms were refined using a riding model. The methyl H atoms were constrained to an ideal geometry (C—H = 0.96 Å) with *U*ĩso~(H)= 1.5Ueq(C), but were allowed to rotate freely about the adjacent C—C bonds. The other H atoms were refined using a riding model, with C—H = 0.97 Å (resp N—H = 0.90 Å) and *U*_iso_(H) = 1.2Ueq(C or N).

### Crystal data

**Table:**

(C_4_H_14_N_2_)_2_[Co(C_2_O_4_)_3_]·2H_2_O	*F*(000) = 1132
*M**_r_* = 539.37	*D*_x_ = 1.537 Mg m^−^^3^
Monoclinic, *P*2_1_/*c*	Mo *K*α radiation, λ = 0.71070 Å
*a* = 12.625 (3) Å	Cell parameters from 5427 reflections
*b* = 13.411 (4) Å	θ = 0.4–27.5°
*c* = 16.996 (2) Å	µ = 0.81 mm^−^^1^
β = 125.91 (2)°	*T* = 293 K
*V* = 2330.7 (11) Å^3^	Cube, pink
*Z* = 4	0.50 × 0.48 × 0.26 mm

### Data collection

**Table:**

Nonius KappaCCD diffractometer	5342 independent reflections
Radiation source: fine-focus sealed tube, Nonius Kappa CCD	4178 reflections with *I* > 2σ(*I*)
graphite	*R*_int_ = 0.018
Detector resolution: 9 pixels mm^-1^	θ_max_ = 27.5°, θ_min_ = 3.4°
φ and ω scans	*h* = −16→16
Absorption correction: multi-scan (*SCALEPACK*; Otwinowski & Minor, 1997)	*k* = −16→17
*T*_min_ = 0.720, *T*_max_ = 0.800	*l* = −21→22
9121 measured reflections	

### Refinement

**Table:**

Refinement on *F*^2^	Primary atom site location: structure-invariant direct methods
Least-squares matrix: full	Secondary atom site location: difference Fourier map
*R*[*F*^2^ > 2σ(*F*^2^)] = 0.047	Hydrogen site location: difference Fourier map
*wR*(*F*^2^) = 0.133	H-atom parameters constrained
*S* = 1.05	*w* = 1/[σ^2^(*F*_o_^2^) + (0.0615*P*)^2^ + 2.554*P*] where *P* = (*F*_o_^2^ + 2*F*_c_^2^)/3
5333 reflections	(Δ/σ)_max_ = 0.001
302 parameters	Δρ_max_ = 1.24 e Å^−^^3^
0 restraints	Δρ_min_ = −0.48 e Å^−^^3^

### Special details

**Table:**

Geometry. All e.s.d.'s (except the e.s.d. in the dihedral angle between two l.s. planes) are estimated using the full covariance matrix. The cell e.s.d.'s are taken into account individually in the estimation of e.s.d.'s in distances, angles and torsion angles; correlations between e.s.d.'s in cell parameters are only used when they are defined by crystal symmetry. An approximate (isotropic) treatment of cell e.s.d.'s is used for estimating e.s.d.'s involving l.s. planes.
Refinement. Refinement of *F*^2^ against ALL reflections. The weighted *R*-factor *wR* and goodness of fit *S* are based on *F*^2^, conventional *R*-factors *R* are based on *F*, with *F* set to zero for negative *F*^2^. The threshold expression of *F*^2^ > σ(*F*^2^) is used only for calculating *R*-factors(gt) *etc*. and is not relevant to the choice of reflections for refinement. *R*-factors based on *F*^2^ are statistically about twice as large as those based on *F*, and *R*- factors based on ALL data will be even larger.

### Fractional atomic coordinates and isotropic or equivalent isotropic displacement parameters (Å^2^)

**Table:**

	*x*	*y*	*z*	*U*_iso_*/*U*_eq_	
Co1	0.19856 (3)	0.25025 (2)	0.04584 (2)	0.02870 (12)	
O1	0.1066 (2)	0.28305 (14)	0.11324 (14)	0.0380 (4)	
O2	0.1676 (2)	0.40241 (14)	0.02043 (14)	0.0373 (4)	
O3	0.38888 (19)	0.27837 (17)	0.17225 (13)	0.0404 (5)	
O4	0.30589 (18)	0.24570 (14)	−0.01047 (13)	0.0341 (4)	
O5	0.19519 (17)	0.09766 (13)	0.06110 (12)	0.0326 (4)	
O6	0.02190 (17)	0.21331 (13)	−0.08801 (13)	0.0342 (4)	
O7	0.0523 (2)	0.41384 (16)	0.16340 (16)	0.0492 (5)	
O8	0.1380 (2)	0.53582 (14)	0.08303 (15)	0.0423 (5)	
O9	0.5759 (2)	0.33994 (19)	0.20796 (14)	0.0520 (6)	
O10	0.4998 (2)	0.2882 (2)	0.02668 (16)	0.0600 (7)	
O11	0.09729 (19)	−0.03816 (13)	−0.02854 (13)	0.0358 (4)	
O12	−0.0938 (2)	0.07973 (15)	−0.17267 (13)	0.0477 (5)	
C1	0.0942 (3)	0.3754 (2)	0.12066 (18)	0.0321 (5)	
C2	0.1363 (3)	0.44434 (19)	0.06991 (18)	0.0319 (5)	
C3	0.4665 (3)	0.3009 (2)	0.15178 (18)	0.0335 (6)	
C4	0.4215 (3)	0.2767 (2)	0.04675 (18)	0.0328 (5)	
C5	0.1060 (2)	0.05329 (18)	−0.01538 (16)	0.0268 (5)	
C6	0.0010 (2)	0.12061 (18)	−0.09993 (17)	0.0288 (5)	
C11	0.4502 (4)	0.9703 (3)	0.1245 (3)	0.0728 (11)	
H11A	0.5370	0.9437	0.1670	0.109*	
H11B	0.4438	1.0308	0.1516	0.109*	
H11C	0.4316	0.9839	0.0621	0.109*	
N10	0.3537 (3)	0.8959 (2)	0.11339 (17)	0.0477 (6)	
H10N	0.3732	0.8813	0.1722	0.057*	
H10M	0.2729	0.9225	0.0771	0.057*	
C12	0.3555 (4)	0.8048 (3)	0.0674 (3)	0.0550 (8)	
H12A	0.4417	0.7746	0.1064	0.066*	
H12B	0.3337	0.8194	0.0035	0.066*	
C13	0.2585 (3)	0.7372 (3)	0.0593 (2)	0.0548 (9)	
H13A	0.1719	0.7653	0.0144	0.066*	
H13B	0.2744	0.7310	0.1222	0.066*	
N9	0.2635 (3)	0.6321 (2)	0.02300 (19)	0.0516 (7)	
H9N1	0.2019	0.5941	0.0192	0.062*	
H9N2	0.2412	0.6386	−0.0377	0.062*	
C14	0.3862 (4)	0.5786 (4)	0.0800 (3)	0.0795 (13)	
H14A	0.4520	0.6153	0.0807	0.119*	
H14B	0.3757	0.5141	0.0518	0.119*	
H14C	0.4122	0.5709	0.1452	0.119*	
O13	0.7540 (2)	0.3562 (2)	0.14165 (16)	0.0632 (7)	
H13O	0.8069	0.3745	0.2082	0.095*	
H13W	0.6669	0.3440	0.1227	0.095*	
O14	0.2551 (4)	0.4143 (3)	0.3690 (2)	0.1119 (14)	
H14O	0.1813	0.4028	0.3031	0.168*	
H14W	0.2973	0.3604	0.4146	0.168*	
N8	−0.1051 (2)	0.12399 (17)	0.28323 (16)	0.0366 (5)	
H8NA	−0.0724	0.1751	0.3256	0.044*	
H8NB	−0.0788	0.0667	0.3173	0.044*	
N7	0.1385 (2)	0.11853 (17)	0.22373 (15)	0.0323 (5)	
H7NA	0.1035	0.0668	0.1821	0.039*	
H7NB	0.1129	0.1752	0.1888	0.039*	
C7	0.2830 (3)	0.1114 (3)	0.2847 (2)	0.0470 (7)	
H7A	0.3200	0.1654	0.3304	0.070*	
H7B	0.3126	0.1149	0.2441	0.070*	
H7C	0.3098	0.0491	0.3191	0.070*	
C8	0.0909 (3)	0.1176 (2)	0.2861 (2)	0.0435 (7)	
H8C	0.1306	0.1720	0.3325	0.052*	
H8D	0.1152	0.0554	0.3218	0.052*	
C9	−0.0551 (3)	0.1289 (2)	0.2227 (2)	0.0431 (7)	
H9A	−0.0790	0.1923	0.1889	0.052*	
H9B	−0.0944	0.0761	0.1745	0.052*	
C10	−0.2507 (3)	0.1290 (3)	0.2196 (2)	0.0522 (8)	
H10A	−0.2781	0.1938	0.1902	0.078*	
H10B	−0.2828	0.1173	0.2579	0.078*	
H10C	−0.2849	0.0792	0.1698	0.078*	

### Atomic displacement parameters (Å^2^)

**Table:**

	*U*^11^	*U*^22^	*U*^33^	*U*^12^	*U*^13^	*U*^23^
Co1	0.02854 (19)	0.02694 (19)	0.02907 (19)	−0.00288 (13)	0.01603 (15)	−0.00256 (13)
O1	0.0469 (11)	0.0303 (9)	0.0484 (11)	−0.0013 (9)	0.0343 (10)	0.0026 (8)
O2	0.0533 (11)	0.0260 (9)	0.0479 (11)	−0.0014 (8)	0.0383 (10)	−0.0007 (8)
O3	0.0336 (10)	0.0596 (13)	0.0273 (9)	−0.0106 (9)	0.0175 (8)	−0.0065 (9)
O4	0.0287 (9)	0.0466 (11)	0.0263 (9)	−0.0050 (8)	0.0157 (7)	−0.0088 (7)
O5	0.0323 (9)	0.0259 (9)	0.0267 (8)	0.0001 (7)	0.0101 (7)	0.0016 (7)
O6	0.0315 (9)	0.0238 (9)	0.0313 (9)	0.0001 (7)	0.0095 (8)	0.0033 (7)
O7	0.0695 (15)	0.0436 (12)	0.0593 (13)	0.0075 (11)	0.0517 (12)	0.0045 (10)
O8	0.0601 (13)	0.0273 (10)	0.0505 (12)	−0.0033 (9)	0.0385 (11)	−0.0035 (8)
O9	0.0340 (11)	0.0808 (17)	0.0327 (10)	−0.0197 (11)	0.0148 (9)	−0.0160 (10)
O10	0.0396 (12)	0.107 (2)	0.0396 (12)	−0.0170 (13)	0.0268 (10)	−0.0131 (12)
O11	0.0424 (10)	0.0249 (9)	0.0337 (9)	0.0000 (8)	0.0188 (8)	0.0001 (7)
O12	0.0432 (11)	0.0347 (11)	0.0292 (10)	−0.0058 (9)	0.0010 (9)	−0.0006 (8)
C1	0.0327 (13)	0.0331 (13)	0.0326 (13)	0.0011 (11)	0.0203 (11)	0.0018 (10)
C2	0.0355 (14)	0.0286 (13)	0.0332 (13)	−0.0005 (10)	0.0210 (11)	0.0006 (10)
C3	0.0303 (13)	0.0386 (15)	0.0258 (12)	−0.0009 (11)	0.0133 (10)	−0.0023 (10)
C4	0.0292 (13)	0.0390 (14)	0.0273 (12)	−0.0012 (11)	0.0150 (11)	−0.0022 (10)
C5	0.0282 (12)	0.0255 (12)	0.0252 (11)	0.0004 (9)	0.0147 (10)	0.0018 (9)
C6	0.0293 (12)	0.0262 (12)	0.0254 (11)	−0.0004 (10)	0.0128 (10)	0.0013 (9)
C11	0.058 (2)	0.071 (3)	0.086 (3)	−0.004 (2)	0.041 (2)	0.003 (2)
N10	0.0509 (15)	0.0483 (15)	0.0315 (12)	0.0179 (12)	0.0171 (11)	0.0057 (10)
C12	0.056 (2)	0.061 (2)	0.0536 (19)	−0.0043 (17)	0.0353 (17)	−0.0044 (16)
C13	0.0442 (18)	0.081 (3)	0.0441 (17)	−0.0077 (16)	0.0286 (15)	−0.0094 (16)
N9	0.0537 (16)	0.0543 (16)	0.0420 (14)	−0.0178 (13)	0.0253 (13)	0.0014 (12)
C14	0.059 (2)	0.085 (3)	0.088 (3)	0.000 (2)	0.040 (2)	0.028 (2)
O13	0.0463 (13)	0.104 (2)	0.0393 (12)	−0.0317 (13)	0.0251 (10)	−0.0199 (12)
O14	0.138 (3)	0.080 (2)	0.077 (2)	−0.014 (2)	0.040 (2)	0.0358 (18)
N8	0.0499 (14)	0.0315 (11)	0.0385 (12)	−0.0079 (10)	0.0315 (11)	−0.0056 (9)
N7	0.0361 (11)	0.0314 (11)	0.0307 (10)	−0.0083 (9)	0.0204 (9)	−0.0060 (9)
C7	0.0385 (15)	0.059 (2)	0.0353 (14)	−0.0051 (14)	0.0171 (12)	0.0006 (13)
C8	0.0480 (16)	0.0519 (18)	0.0325 (14)	−0.0062 (14)	0.0248 (13)	−0.0056 (12)
C9	0.0486 (17)	0.0490 (17)	0.0361 (14)	0.0001 (14)	0.0273 (13)	0.0006 (12)
C10	0.0488 (18)	0.055 (2)	0.0487 (18)	0.0006 (15)	0.0261 (15)	0.0021 (15)

### Geometric parameters (Å, °)

**Table:**

Co1—O5	2.0663 (19)	C13—H13A	0.9700
Co1—O4	2.0680 (19)	C13—H13B	0.9700
Co1—O2	2.075 (2)	N9—C14	1.446 (5)
Co1—O1	2.1004 (19)	N9—H9N1	0.9000
Co1—O6	2.1079 (19)	N9—H9N2	0.9000
Co1—O3	2.116 (2)	C14—H14A	0.9600
O1—C1	1.264 (3)	C14—H14B	0.9600
O2—C2	1.253 (3)	C14—H14C	0.9600
O3—C3	1.254 (3)	O13—H13O	0.9484
O4—C4	1.259 (3)	O13—H13W	0.9606
O5—C5	1.263 (3)	O14—H14O	0.9599
O6—C6	1.262 (3)	O14—H14W	0.9599
O7—C1	1.235 (3)	N8—C9	1.490 (3)
O8—C2	1.245 (3)	N8—C10	1.490 (4)
O9—C3	1.243 (3)	N8—H8NA	0.9000
O10—C4	1.229 (3)	N8—H8NB	0.9000
O11—C5	1.240 (3)	N7—C7	1.480 (4)
O12—C6	1.235 (3)	N7—C8	1.494 (3)
C1—C2	1.555 (3)	N7—H7NA	0.9000
C3—C4	1.557 (3)	N7—H7NB	0.9000
C5—C6	1.550 (3)	C7—H7A	0.9600
C11—N10	1.498 (5)	C7—H7B	0.9600
C11—H11A	0.9600	C7—H7C	0.9600
C11—H11B	0.9600	C8—C9	1.501 (4)
C11—H11C	0.9600	C8—H8C	0.9700
N10—C12	1.458 (4)	C8—H8D	0.9700
N10—H10N	0.9000	C9—H9A	0.9700
N10—H10M	0.9000	C9—H9B	0.9700
C12—C13	1.463 (5)	C10—H10A	0.9600
C12—H12A	0.9700	C10—H10B	0.9600
C12—H12B	0.9700	C10—H10C	0.9600
C13—N9	1.555 (5)		
			
O5—Co1—O4	95.52 (8)	C12—C13—N9	111.9 (3)
O5—Co1—O2	170.01 (8)	C12—C13—H13A	109.2
O4—Co1—O2	91.57 (7)	N9—C13—H13A	109.2
O5—Co1—O1	94.60 (7)	C12—C13—H13B	109.2
O4—Co1—O1	168.55 (7)	N9—C13—H13B	109.2
O2—Co1—O1	79.08 (7)	H13A—C13—H13B	107.9
O5—Co1—O6	79.40 (7)	C14—N9—C13	117.4 (3)
O4—Co1—O6	93.41 (8)	C14—N9—H9N1	107.9
O2—Co1—O6	93.19 (8)	C13—N9—H9N1	107.9
O1—Co1—O6	93.70 (8)	C14—N9—H9N2	107.9
O5—Co1—O3	98.27 (8)	C13—N9—H9N2	107.9
O4—Co1—O3	79.23 (7)	H9N1—N9—H9N2	107.2
O2—Co1—O3	89.95 (9)	N9—C14—H14A	109.5
O1—Co1—O3	94.04 (8)	N9—C14—H14B	109.5
O6—Co1—O3	172.08 (8)	H14A—C14—H14B	109.5
C1—O1—Co1	113.60 (16)	N9—C14—H14C	109.5
C2—O2—Co1	113.38 (16)	H14A—C14—H14C	109.5
C3—O3—Co1	111.60 (16)	H14B—C14—H14C	109.5
C4—O4—Co1	114.24 (16)	H13O—O13—H13W	108.1
C5—O5—Co1	114.15 (15)	H14O—O14—H14W	121.4
C6—O6—Co1	113.20 (15)	C9—N8—C10	109.8 (2)
O7—C1—O1	126.1 (2)	C9—N8—H8NA	109.7
O7—C1—C2	118.8 (2)	C10—N8—H8NA	109.7
O1—C1—C2	115.1 (2)	C9—N8—H8NB	109.7
O8—C2—O2	125.7 (2)	C10—N8—H8NB	109.7
O8—C2—C1	117.5 (2)	H8NA—N8—H8NB	108.2
O2—C2—C1	116.8 (2)	C7—N7—C8	110.2 (2)
O9—C3—O3	125.8 (2)	C7—N7—H7NA	109.6
O9—C3—C4	117.5 (2)	C8—N7—H7NA	109.6
O3—C3—C4	116.7 (2)	C7—N7—H7NB	109.6
O10—C4—O4	125.7 (2)	C8—N7—H7NB	109.6
O10—C4—C3	118.7 (2)	H7NA—N7—H7NB	108.1
O4—C4—C3	115.6 (2)	N7—C7—H7A	109.5
O11—C5—O5	125.7 (2)	N7—C7—H7B	109.5
O11—C5—C6	118.1 (2)	H7A—C7—H7B	109.5
O5—C5—C6	116.2 (2)	N7—C7—H7C	109.5
O12—C6—O6	125.9 (2)	H7A—C7—H7C	109.5
O12—C6—C5	118.0 (2)	H7B—C7—H7C	109.5
O6—C6—C5	116.1 (2)	N7—C8—C9	109.1 (2)
N10—C11—H11A	109.5	N7—C8—H8C	109.9
N10—C11—H11B	109.5	C9—C8—H8C	109.9
H11A—C11—H11B	109.5	N7—C8—H8D	109.9
N10—C11—H11C	109.5	C9—C8—H8D	109.9
H11A—C11—H11C	109.5	H8C—C8—H8D	108.3
H11B—C11—H11C	109.5	N8—C9—C8	109.8 (2)
C12—N10—C11	111.0 (3)	N8—C9—H9A	109.7
C12—N10—H10N	109.4	C8—C9—H9A	109.7
C11—N10—H10N	109.4	N8—C9—H9B	109.7
C12—N10—H10M	109.4	C8—C9—H9B	109.7
C11—N10—H10M	109.4	H9A—C9—H9B	108.2
H10N—N10—H10M	108.0	N8—C10—H10A	109.5
N10—C12—C13	107.1 (3)	N8—C10—H10B	109.5
N10—C12—H12A	110.3	H10A—C10—H10B	109.5
C13—C12—H12A	110.3	N8—C10—H10C	109.5
N10—C12—H12B	110.3	H10A—C10—H10C	109.5
C13—C12—H12B	110.3	H10B—C10—H10C	109.5
H12A—C12—H12B	108.6		
			
O5—Co1—O1—C1	177.89 (18)	Co1—O1—C1—O7	−174.5 (2)
O4—Co1—O1—C1	25.8 (5)	Co1—O1—C1—C2	5.8 (3)
O2—Co1—O1—C1	−9.93 (18)	Co1—O2—C2—O8	164.7 (2)
O6—Co1—O1—C1	−102.47 (19)	Co1—O2—C2—C1	−14.1 (3)
O3—Co1—O1—C1	79.24 (19)	O7—C1—C2—O8	7.1 (4)
O5—Co1—O2—C2	64.5 (5)	O1—C1—C2—O8	−173.2 (2)
O4—Co1—O2—C2	−160.22 (19)	O7—C1—C2—O2	−174.1 (3)
O1—Co1—O2—C2	13.13 (18)	O1—C1—C2—O2	5.7 (4)
O6—Co1—O2—C2	106.28 (19)	Co1—O3—C3—O9	164.8 (3)
O3—Co1—O2—C2	−80.99 (19)	Co1—O3—C3—C4	−15.7 (3)
O5—Co1—O3—C3	108.9 (2)	Co1—O4—C4—O10	−174.0 (3)
O4—Co1—O3—C3	14.76 (19)	Co1—O4—C4—C3	6.7 (3)
O2—Co1—O3—C3	−76.8 (2)	O9—C3—C4—O10	6.8 (4)
O1—Co1—O3—C3	−155.9 (2)	O3—C3—C4—O10	−172.7 (3)
O6—Co1—O3—C3	36.6 (7)	O9—C3—C4—O4	−173.8 (3)
O5—Co1—O4—C4	−108.70 (19)	O3—C3—C4—O4	6.6 (4)
O2—Co1—O4—C4	78.35 (19)	Co1—O5—C5—O11	167.8 (2)
O1—Co1—O4—C4	43.4 (5)	Co1—O5—C5—C6	−10.8 (3)
O6—Co1—O4—C4	171.64 (19)	Co1—O6—C6—O12	−178.4 (2)
O3—Co1—O4—C4	−11.30 (19)	Co1—O6—C6—C5	0.7 (3)
O4—Co1—O5—C5	−83.76 (17)	O11—C5—C6—O12	7.4 (4)
O2—Co1—O5—C5	51.3 (5)	O5—C5—C6—O12	−173.9 (2)
O1—Co1—O5—C5	101.59 (18)	O11—C5—C6—O6	−171.7 (2)
O6—Co1—O5—C5	8.69 (17)	O5—C5—C6—O6	7.0 (3)
O3—Co1—O5—C5	−163.64 (17)	C11—N10—C12—C13	−179.8 (3)
O5—Co1—O6—C6	−4.67 (17)	N10—C12—C13—N9	−172.8 (3)
O4—Co1—O6—C6	90.32 (18)	C12—C13—N9—C14	58.1 (4)
O2—Co1—O6—C6	−177.91 (18)	C7—N7—C8—C9	177.9 (3)
O1—Co1—O6—C6	−98.67 (18)	C10—N8—C9—C8	−176.6 (3)
O3—Co1—O6—C6	68.9 (6)	N7—C8—C9—N8	177.7 (2)

### Hydrogen-bond geometry (Å, °)

**Table:**

*D*—H···*A*	*D*—H	H···*A*	*D*···*A*	*D*—H···*A*
N9—H9N1···O8	0.90	1.86	2.668 (3)	148.
O14—H14O···O7	0.96	1.94	2.876 (4)	163.
N7—H7NB···O1	0.90	1.90	2.769 (3)	161.
O13—H13W···O10	0.96	1.91	2.755 (3)	146.
N10—H10M···O11^i^	0.90	1.94	2.814 (3)	165.
N10—H10N···O9^ii^	0.90	1.83	2.721 (3)	173.
N9—H9N2···O13^iii^	0.90	1.80	2.679 (3)	163.
N8—H8NA···O6^iv^	0.90	1.94	2.829 (3)	170.
N8—H8NB···O7^v^	0.90	2.07	2.916 (3)	156.
N8—H8NB···O8^v^	0.90	2.25	2.801 (3)	119.
N7—H7NA···O12^vi^	0.90	1.97	2.751 (3)	144.
O13—H13O···O12^vii^	0.95	1.75	2.698 (3)	174.

Symmetry codes: (i) *x*, *y*+1, *z*; (ii) −*x*+1, *y*+1/2, −*z*+1/2; (iii) −*x*+1, −*y*+1, −*z*; (iv) *x*, −*y*+1/2, *z*+1/2; (v) −*x*, *y*−1/2, −*z*+1/2; (vi) −*x*, −*y*, −*z*; (vii) *x*+1, −*y*+1/2, *z*+1/2.
